# Rapid transformation of heterocyclic building blocks into nanoporous carbons for high-performance supercapacitors†

**DOI:** 10.1039/c8ra00546j

**Published:** 2018-04-03

**Authors:** Babak Ashourirad, Muslum Demir, Ryon A. Smith, Ram B. Gupta, Hani M. El-Kaderi

**Affiliations:** Department of Chemistry, Virginia Commonwealth University Richmond VA 23284 USA ashouriradb@vcu.edu helkaderi@vcu.edu +1 804 828 8599 +1 804 828 7505; Department of Chemical and Life Science Engineering, Virginia Commonwealth University Richmond VA 23284 USA; Department of Chemical Engineering, Osmaniye Korkut Ata University 80000 Osmaniye Turkey

## Abstract

The ever-increasing global energy consumption necessitates the development of efficient energy conversion and storage devices. Nitrogen-doped porous carbons as electrode materials for supercapacitors feature superior electrochemical performances compared to pristine activated carbons. Herein, a facile synthetic strategy including solid-state mixing of benzimidazole as an inexpensive single-source precursor of nitrogen and carbon and zinc chloride as a high temperature solvent/activator followed by pyrolysis of the mixture (*T* = 700–1000 °C under Ar) is introduced. The addition of ZnCl_2_ prevents early sublimation of benzimidazole and promotes carbonization and pore generation. The sample obtained under the optimal carbonization temperature of 900 °C and ZnCl_2_/benzimidazole weight ratio of 2/1 (ZBIDC-2-900) features a moderate specific surface area of 855 m^2^ g^−1^, high N-doping level (10 wt%), and a wide micropore size distribution (∼1 nm). ZBIDC-2-900 as a supercapacitor electrode exhibits a large gravimetric capacitance of 332 F g^−1^ (at 1 A g^−1^ in 1 M H_2_SO_4_) thanks to the cooperative advantages of the electrochemical activity of the nitrogen functional groups and the accessible porosity. The excellent capacitance performance coupled with robust cyclic stability, high yield and straightforward synthesis of the proposed carbons holds great potential for large-scale energy storage applications.

## Abstract 2

Zinc chloride activated benzimidazole derived carbons (ZBIDCs) with optimal textural and chemical properties exhibit remarkable and stable performance in supercapacitor applications.

## Graphical abstract

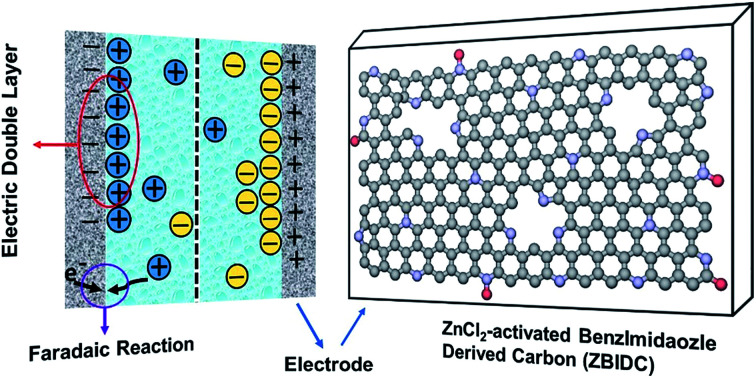

## 1. Introduction

Explosive growth in the global energy demand and consumption over the last decade has posed an imminent threat to future generations. Currently, the power network stability is managed through balancing the load of fossil fuel plants. To reduce the reliance on fossil fuels and mitigate the CO_2_ emissions, the effective utilization of clean renewable energy is regarded as an absolute necessity.^1^ However, the intermittent nature of most renewable energy sources such as wind and solar is a major challenge for maintaining a stable power flow. In this context, the development of various types of energy storage devices with the ability to efficiently store/release energy is imperative. The use of electrochemical supercapacitors as promising energy storage devices has drawn immense attention due to their high power density, fast charge/discharge rate, long cycle lifetime, and wide range of operating temperatures.^2^

According to their energy storage mechanism, supercapacitors can be classified as electrical double layer capacitors (EDLCs) and pseudocapacitors. EDLCs store energy based on charge accumulation along the double layer formed at the electrode–electrolyte interface while pseudocapacitors store energy through reversible faradaic redox reactions at the surface of electrode materials.^3,4^ Activated carbons (ACs) are predominantly used as the electrode materials for commercial EDLCs due to their large surface area and adequate pore size, which are basic requirements for creating accessible paths for ionic transport and double layer formation.^5,6^ Additionally, ACs feature exceptional properties such as high electronic conductivity, excellent physiochemical stability, wide availability of raw materials, easy manufacturing processes and controllable surface chemistry.^7,8^ The latter feature is of particular importance because the electronic distribution of plain carbons can be positively modified by the incorporation of heteroatom species.^9^ For instance, oxygen functionalities usually found on the surface of activated carbons generally feature acidic aspects and as such promote electron–acceptor behavior. On the contrary, the basic nature of nitrogen surface groups endows the carbon framework with electron–donor characteristics.^10^ Among all heteroatoms, nitrogen is the most frequently studied dopant due to its versatility, availability and ease of incorporation methods into the carbon backbone.^11^ It has been shown that nitrogen incorporation gives rise to the overall capacitance through inducing pseudocapacitance with faradaic reactions as well as enhancing the wettability (towards aqueous electrolytes) and electron conductivity of carbon-based electrodes.^12^ The accessible pyrrolic and pyridinic nitrogen entities provide chemically active sites, which have a more pronounced effect on the capacitive performance.^13,14^ Therefore, nitrogen-doped porous carbons (NDPCs) are promising candidates for energy storage applications.

Two main strategies have been adopted to incorporate nitrogen into carbon matrices. The first approach includes the thermal treatment of plain activated carbons with an external nitrogen-rich source (*i.e.* ammonia, amine, aniline, nitric acid, pyrrole or pyridine), whereas in the second method *in situ* doping takes place, using two different reagents for nitrogen and carbon followed by a subsequent carbonization–activation process.^15^ The post-treatment method suffers from critical drawbacks such as low and non-homogeneous nitrogen doping, toxic and corrosive precursors and tedious procedures.^16^ Consequently, *in situ* doping is of particular interest especially if a single reagent containing both carbon and nitrogen is used as a precursor. Accordingly, a wide spectrum of single-source precursors has been successfully converted into NDPCs. Recently, synthetic polymers have been extensively transformed into NDPCs with uniform doping using one-step chemical activation or direct carbonization.^17–20^ However, drawbacks such as the need for multi-step reactions, the use of toxic organic solvents, low polymer yields and high overall costs are slowing down the adoption of this synthetic strategy. The use of plentiful and renewable biomass sources or biomass derivatives as the sole precursors has overcome the high cost problem and favors sustainability.^21,22^ Nevertheless, the use of biomass initially requires processing steps such as cleaning, drying, pulverizing, hydrothermal carbonization and impregnation with desirable porogens before carbonization.^23–25^ More importantly, the carbon products obtained by activation of these bio-chars exhibit inconsistent electrochemical properties, which is detrimental for their use in energy storage applications. All-in-one precursors composed of ionic liquids (ILs) or organic salts^26,27^ are able to provide homogeneous nitrogen doping through direct pyrolysis without the need for an extra activation step.^28–31^ In spite of the facile carbonization of ILs and salts, the design and synthesis of their nitrogen-containing components and the limited structural control over the final carbon product remain as major challenges. The use of nitrogen-containing organic molecules which are able to undergo carbonization without requiring initial polymerization is considered an effective solution for the aforementioned problems. Mokaya and coworkers fabricated a series of graphitic NDPCs by using acetonitrile as the precursor and applying chemical vapor deposition (CVD) using various silica templates.^32^ The high energy needed for the cyclization–aromatization of linear acetonitrile is provided by the costly CVD method. Moreover, the template methods are somewhat complex and require several steps including pre-fabrication and removal of the templates. Consequently, the development of facile carbonization methodology is as equally important as selecting a proper single-source precursor. Overcoming all of these challenges is conceivable if a heterocyclic building block is used as the sole precursor and a one-step carbonization/activation procedure is adopted as the synthetic strategy.

Herein, we utilize benzimidazole, a heterocyclic building block with 25 wt% nitrogen content, as the sole precursor of carbon and nitrogen. The intrinsic aromatic structure and arrangement of nitrogen atoms at the pyridinic and pyrrolic positions in benzimidazole support the formation of graphitic nitrogen-doped carbon with a minimum driving force. The straightforward, one-step and solvent-free synthetic reaction involves physical mixing of benzimidazole with zinc chloride followed by pyrolysis at high temperatures. The multifaceted roles of zinc chloride in complex formation, facilitation of the polymerization–carbonization processes and pore generation are studied. The zinc chloride activated benzimidazole derived carbons (ZBIDCs) feature a modest surface area, high nitrogen-doping levels and a suitable degree of graphitization. It was found that variation of the synthesis temperature could be used as a tool to precisely control the porous texture and surface chemistry of the ZBIDCs while these properties remained unaffected by altering the amount of ZnCl_2_. We further evaluated the electrochemical performance of the ZBIDCs as electrode materials for supercapacitor applications. The resultant carbons offer superior capacitive behavior because of the cooperative effects of the electric double layer and faradaic transitions. The solvent- and template-free nature of our proposed synthetic procedure coupled with the extremely low price and commercial availability of benzimidazole and ZnCl_2_ reagents promote an environmentally friendly and scalable production method. Furthermore, the high yield, desirable electrochemical performance and robust cyclic stability of the ZBIDCs suggest potential advantages for the industrialized application of supercapacitors.

## 2. Experimental

### 2.1. Materials and synthesis

All of the chemicals used in this work were commercial analytical reagents and were used without any further purification. ZnCl_2_ (Alfa Aesar, anhydrous, >98%) and benzimidazole (TCI America, >98%) were stored in a glovebox and used as received. To minimize the effect of ambient moisture, selected ratios of as received benzimidazole (BI) and ZnCl_2_ were mixed inside a glovebox by grinding using a mortar and pestle prior to carbonization. In the first control, 300 mg of BI was thoroughly mixed with 600 mg of ZnCl_2_ (2 : 1 weight ratio of activator to precursor) and then transferred to a temperature programmed tube furnace. The freshly prepared white powdery mixture was kept for 1 h under Ar flow at room temperature to remove any traces of air and was then heated to the target temperatures of 700, 800, 900 and 1000 °C at a ramp rate of 5 °C min^−1^ and held for 1 h. In another control, 300 mg of the BI precursor was mixed with 300, 900 and 1200 mg of ZnCl_2_ (1 : 1, 3 : 1 and 4 : 1 weight ratio of activator to precursor) and carbonized in the same fashion at a fixed temperature of 900 °C. A calcination temperature of 700 °C (the boiling point of ZnCl_2_ is 732 °C) as well as a minimum weight BI to ZnCl_2_ ratio of 2 to 1 (an excess amount of ZnCl_2_ is also needed for effective activation) was selected to ensure the simultaneous conversion of precursor to carbon and pore formation. After cooling to room temperature, the black carbon products were soaked and washed three times with 2.0 M HCl to remove metals and residual salts. Further purification was performed by washing the as-synthesized carbons with distilled water followed by ethanol. The obtained zinc-chloride activated benzimidazole derived carbons were denoted as “ZBIDC-*x-y*,” where “*x*” indicates the ZnCl_2_ to BI weight ratio and “*y*” represents the activation temperature. The resulting activated carbons were outgassed under vacuum at 200 °C for 12 h prior to gas sorption measurements.

### 2.2. Characterization techniques

Scanning electron microscopy (SEM) images were obtained using a Hitachi SU-70 scanning electron microscope. The samples were prepared by dispersing each specimen onto the surface of a sticky carbon attached to a flat aluminum sample holder. Then the samples were coated with platinum at a pressure of 10^−5^ mbar in an N_2_ atmosphere for 60 s prior to SEM imaging. Powder X-ray diffraction patterns of the dried samples were collected at room temperature on a Panalytical X’Pert Pro Multipurpose Diffractometer (MPD). The samples were mounted on a zero background sample holder measured in transmission mode using Cu Kα radiation with a 2*θ* range of 5 to 55°. Elemental analyses of carbon, nitrogen, hydrogen, oxygen, and ash were performed at Midwest Microlab, LLC using an Exeter CE440 analyzer. The CHN and O levels were analyzed using combined static/dynamic combustion and Unterzaucher methods, respectively. The Raman spectra were obtained using a Thermo Scientific DXR SmartRaman spectrometer operating at an excitation wavelength of 532 nm. X-ray photoelectron spectroscopy (XPS) analysis was performed on a Thermo Fisher Scientific ESCALAB 250 spectrometer employing an Al Kα (1486.68 eV) X-ray source equipped with a hemispherical analyzer. The samples were prepared for XPS measurements by pressing the carbon specimen into a piece of indium foil, which was then mounted onto the sample holder using double-sided sticky tape. During XPS analysis, a combination of a low-energy electron flood gun and an argon ion flood gun was utilized for charge compensation. The binding energy scale was calibrated by setting the C 1s peak at 285.0 eV. The XPS results were analyzed with the Thermo Avantage software (v4.84). Gas adsorption–desorption measurements for Ar (87 K), N_2_ (77 K), and CO_2_ (273 K) were carried out on an Autosorb-iQ2 volumetric adsorption analyzer (Quantachrome Instruments) using ultrahigh purity grade adsorbates. The specific surface area of the samples was calculated using the Brunauer–Emmett–Teller (BET) method from Ar and N_2_ isotherms. Incremental pore size distributions (PSD) were obtained from the equilibrium branch of Ar (87 K) and/or N_2_ (77 K) isotherms by applying the quench solid density functional theory (QSDFT) model and assuming slit-pore geometry on the carbon material. Ultrafine (<0.7 nm) porosities were investigated using CO_2_ (273 K) isotherms and applying the nonlocal density functional theory (NLDFT) model under similar assumptions. Prior to any adsorption analyses, the samples were degassed at 200 °C for 12 h.

### 2.3. Electrochemical measurements

The electrochemical performances of the ZBIDCs as active supercapacitor electrode materials were investigated by means of cyclic voltammetry (CV), galvanostatic charge–discharge measurements (GCD) and electrochemical impedance spectroscopy (EIS) on a CHI 660E electrochemical workstation (CH Instruments, Inc.) at room temperature. The working electrodes were fabricated by mixing 80 wt% active electrode material, 10 wt% carbon black (Alfa Aesar), and 10 wt% binder (polytetrafluoroethylene: PTFE, 60 wt% dispersion in H_2_O, Aldrich) until a slurry with proper viscosity was obtained. The viscous slurry was cast onto a current collector (titanium foam, 1.5 cm × 3 cm) and dried at 80 °C for 12 h in a vacuum. The dried electrodes were then uniaxially pressed under a weight of 5 ton in order to achieve good electronic contact. The geometric surface area of the prepared electrode was 0.32 cm^2^. The mass loading of the active material in the electrode ranged from 4 to 6 mg cm^−2^. All of the electrochemical measurements were conducted in 1 M H_2_SO_4_ aqueous solution using a three-electrode configuration equipped with the as-prepared ZBIDCs (working electrodes), Pt wires (auxiliary electrodes) and Ag/AgCl (1 M KCl solution as reference electrodes). The voltage range for the CV measurements was −0.5 to 0.5 V (*vs.* Ag/AgCl) at different scan rates of 5, 10, 20, 50, 80 and 100 mV s^−1^. Galvanostatic charge–discharge tests were performed at various current densities of 1, 2, 5, 8, 10, 15, and 20 A g^−1^ within the potential range of −0.5 to 0.5 V (*vs.* Ag/AgCl) with data intervals of 0.1 seconds. The EIS data was collected in a frequency range of 0.01 Hz to 500 kHz with a 5 mV AC amplitude. The series resistance *R*_s_ value is measured based on the starting point of a Nyquist plot (intersection to *X* and *Y*-axis). Two-electrode cell testing was conducted in a 2025 coin cell with two nearly identical electrodes and Fisher brand (P8) filter paper as a separator. 1 M H_2_SO_4_ aqueous solution was used as the electrolyte, within the potential range of 0.0 to 1.0 V. The following equations were used to calculate the gravimetric specific capacitance (*C*_s_, F g^−1^) from the GCD curves:1
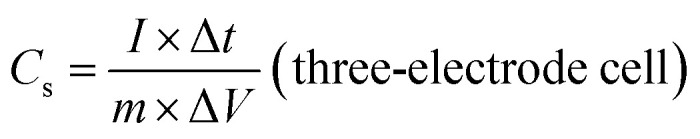
2
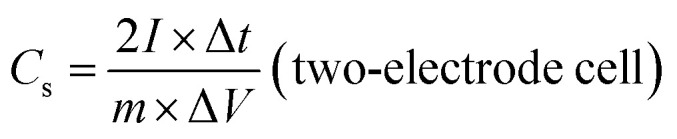
where *I* (A), Δ*t* (s), Δ*V* (V) and *m* (g) represent the discharge current, discharge time, discharging voltage and the mass of active material, respectively.

## 3. Results and discussion

### 3.1. Synthetic approach and structural properties

**Scheme 1:**
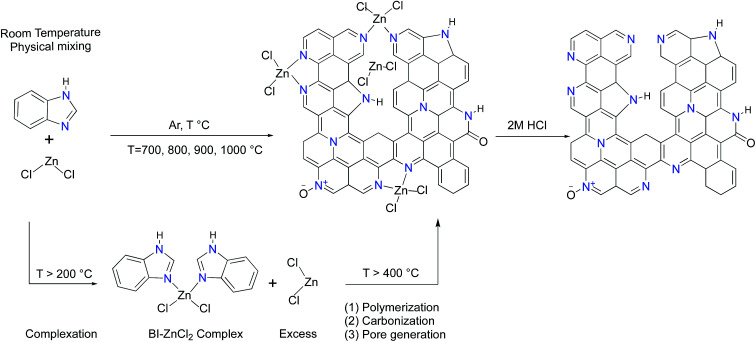
Schematic illustration for the synthesis of ZBIDCs.

It has been well-documented that ZnCl_2_ as a pore forming agent initiates the activation process by promoting structural dehydration if biomass is used as a carbon precursor.^33^ However, further pore development is usually inhibited due to the reaction of ZnCl_2_ with the precursor after the initial dehydration. Accordingly, ZnCl_2_ activation of hydrothermally pretreated biomass precursors leads to the highest surface area and pore volume at 500 °C.^34,35^ It should be mentioned that benzimidazole (BI, hereafter) sublimes if heated by itself while its complexation with ZnCl_2_ dramatically suppresses its premature sublimation.^36^ In contrast to the activation process of the biomass precursors by ZnCl_2_, the BI–ZnCl_2_ mixture at 500 °C yields a uniform molten phase without notable carbonization. To explain how ZnCl_2_ activates BI, a plausible mechanism focusing on the multiple roles of ZnCl_2_ in the reaction mixture must be provided. The primary function of ZnCl_2_ at low temperatures is to bridge the BI building blocks and form a complex^37^ as shown in Scheme 1. Contrary to volatile BI, the *in situ* formed BI–ZnCl_2_ complex has the capacity to endure further high temperature calcination without notable sublimation. As shown in Scheme 1, a minimum stoichiometric BI to ZnCl_2_ molar ratio of 2/1 is needed to achieve the maximal transformation of precursor to carbon. However, the excess amount of ZnCl_2_ salt should be taken into consideration for later polymerization and pore formation steps. At temperatures above 300 °C (ZnCl_2_ melts at 290 °C), the condensed BI–ZnCl_2_ complex forms a homogeneous mixture together with the excess amounts of ZnCl_2_ which were initially designed to be present in the system. As the temperature increases, the excess ZnCl_2_ not only retains its important role as a reaction medium but also acts as a catalyst for the polymerization.^38,39^ Antonietti *et al.* suggested that the binary salt systems (ZnCl_2_ mixed with other chlorides) act as localized templates and leaving groups for pore formation during the carbonization of ionic liquids.^40^ In the case of a single salt system as in the present work, the formation of molten ZnCl_2_ nanodroplets inside the carbonizable BI–ZnCl_2_ complex and later separation from the carbon matrix can be regarded as the primary driving force for pore formation. The pore generation process further proceeds *via* carbothermal reduction of the Zn^2+^ ions at elevated temperatures.^41^ Finally, removal of the chemicals trapped inside the pores of the newly formed carbon *via* acid washing results in the ultimate porous structure.

**Fig. 1:**
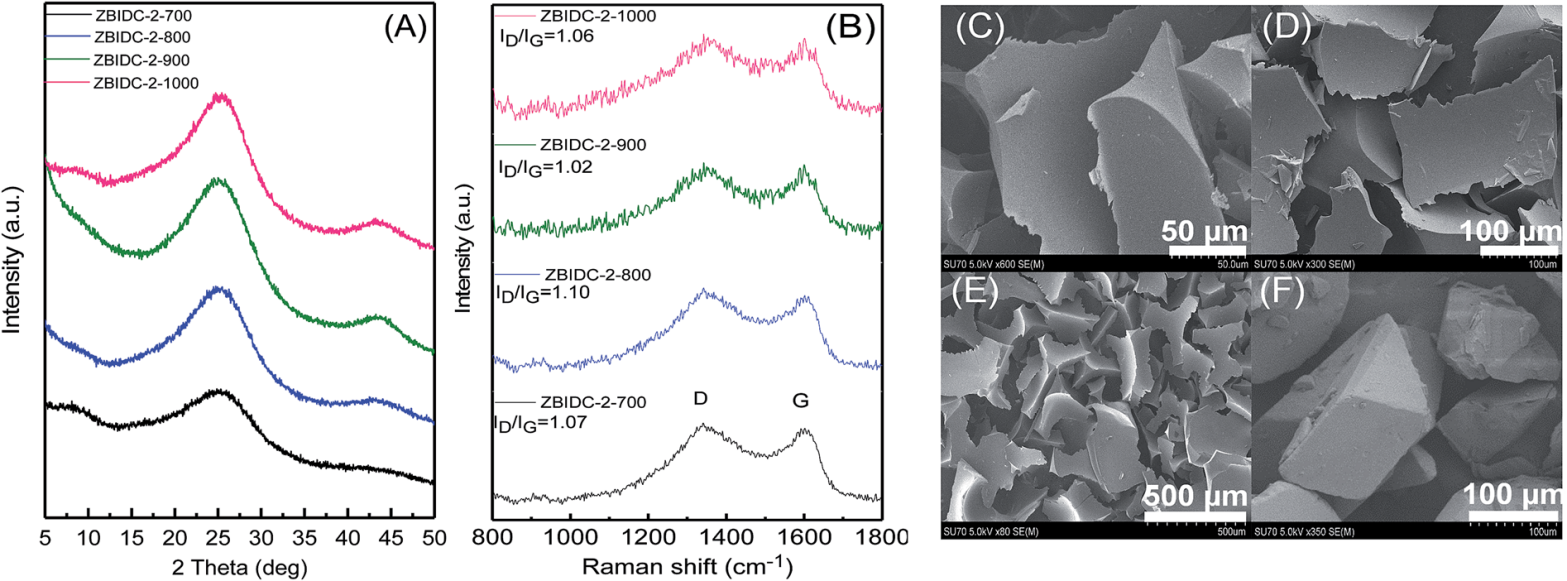
(A) XRD patterns and (B) Raman spectra for the ZBIDCs prepared at various temperatures. Scanning electron microscopy (SEM) images of ZBIDC-2-900 at (C) 50 μm, (D) 100 μm and (E) 500 μm, and (F) of the benzimidazole precursor at 100 μm.

The structures of the ZBIDCs were analyzed by XRD, Raman spectroscopy, and SEM (Fig. 1). As shown in Fig. 1A, the XRD pattern of the ZBIDCs displayed distinct peaks centered at 25 and 43 which are indexed to the (002) and (100) planes of the graphitic layers, respectively.^42^ The absence of any additional peaks indicates the effective removal of any possible salt and/or metallic phases during the previously described washing process. The broad and low-intensity diffraction peaks observed in this study suggest a less-ordered stacking of graphite layers due to the smaller graphitic regions and the local distortion of the carbon lattice by nitrogen incorporation. In general, high activation temperatures provide the necessary driving force for the formation of more crystalline domains and promote a higher degree of graphitization. As shown in Fig. 1B, the Raman spectra feature two characteristic D-band and G-band peaks at 1340 and 1600 cm^−1^, respectively. The D-band results from structural defects and partially disordered structures whereas the G-band originates from sp^2^-hybridized graphitic carbon atoms.^43^ The ratio of the D-band to the G-band intensities (*I*_D_/*I*_G_) is commonly interpreted as a disorder degree of the carbon matrix. All of the samples displayed similar *I*_D_/*I*_G_ values near to unity, which suggests the existence of both ordered and disordered carbon domains. Nevertheless, the lowest *I*_D_/*I*_G_ observed for carbon synthesized at 900 °C indicates a higher degree of graphitization with respect to other carbons. The results from the Raman spectra and XRD patterns of the ZBIDCs indicate a partial graphitic order with crystalline domains, which promises good conductivity. The SEM images of ZBIDC-2-900 as a representative carbon at different magnifications together with the BI precursor are presented in Fig. 1C–F. The microstructure of the carbon sample consisted of large plates featuring high degrees of irregularity, rough surfaces, diverse thicknesses and entirely different shapes from the large blocks of the parent BI. The development of this morphology was probably initiated by the formation of a uniform BI–ZnCl_2_ melt during the early stages of the heat treatment, followed by nucleation of the new phase from the molten media at higher temperatures.

### 3.2. Textural properties and compositional studies

**Fig. 2:**
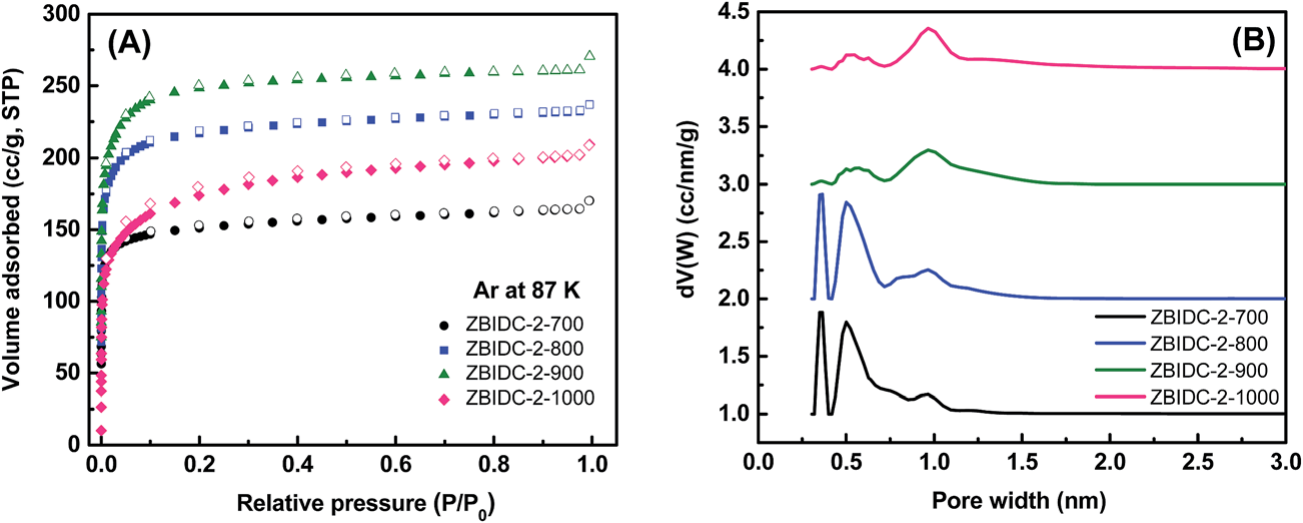
(A) Argon isotherms at 87 K (solid symbols for adsorption and open symbols for desorption) and (B) pore size distributions calculated using the DFT method for the ZBIDC-2-*y* samples (all PSD curves are offset vertically in steps of 1.0 for clarity).

The Ar (87 K) and N_2_ (77 K) adsorption–desorption isotherms were collected to assess the porous parameters of the ZBIDCs. It is noteworthy that Ar was chosen over N_2_ as the IUPAC recommended adsorbate for assessing microporous systems. This is because the quadrupole moment of the N_2_ molecules can interact with a variety of surface heterogeneities and can lead to a possible change in the micropore filling pressure and inaccurate micropore size distribution.^44^ The Ar (87 K) adsorption isotherms and the corresponding pore size distribution (PSD) curves of the ZBIDCs are depicted in Fig. 2. According to the very recently published IUPAC classification system,^44^ all of the studied ZBIDCs displayed type I(b) isotherms which are featured by a steep uptake within the very low partial pressure region and then plateau for the rest of the pressure range. The summarized results in Table 1 show that all of the carbon materials present a moderate Brunauer–Emmett–Teller (BET) surface area and total pore volume in the range of 525–855 m^2^ g^−1^ and 0.21–0.33 cm^3^ g^−1^, respectively. The surface area and pore volume of the obtained carbons increased with an increase in the activation temperature up to 900 °C suggesting the effectiveness of the activation process. At the higher temperature of 1000 °C, the collapse of the porous architecture resulted in a lower surface area and pore volume. The pore size distribution curves of the ZBIDCs were realized to be entirely confined to diameters below 2 nm with three prominent peaks centered around 0.3, 0.5 and 1 nm. It was also observed that the intensity of the peaks representing the ultrafine pores (0.3 and 0.5 nm) diminished as the temperature of activation increased while the 1 nm peak became broader and more intense. The wider distribution of pores with a 1 nm size in ZBIDC-2-900 compared to ZBIDC-2-1000 can be correlated to the pore shrinkage of the latter. It should be noted that a wide distribution of micropores larger than 0.5 nm could be electrochemically accessible to the aqueous electrolyte ions.^45,46^ To investigate the effect of the ZnCl_2_ amount on the porosity parameters, three samples were synthesized by varying ZnCl_2_/BI to 1, 3 and 4 at a fixed temperature of 900 °C which was previously found to yield the optimum surface area and pore size distribution. To compare the porosity levels, the Ar isotherms of these carbons were collected and the results, along with those for ZBIDC-2-900, are presented in Fig. S1A and B and Table S1.† ZBIDC-3-900 and ZBIDC-4-900 featured almost similar BET surface areas and pore volumes to ZBIDC-2-900. The very low surface area and pore volume realized for ZBIDC-1-900 indicate that the amount of ZnCl_2_ used was large enough to merely generate the carbon material, but not sufficient enough to develop the porosity. The microporous nature of ZBIDC-3-900 and ZBIDC-4-900 contradicts the results of other literature studies implying that higher ratios of ZnCl_2_ to precursor may promote the formation of mesopores.^40,47^ It is understood that the temperature of activation is a key factor in controlling the porous parameters while the ZnCl_2_ to precursor ratio plays a marginal role in the pore development of ZBIDCs. To ensure a fair comparison, the N_2_ (77 K) isotherms along with their derived pore size distribution curves and the porosity parameters of the carbons are also collected and shown in Fig. S1C–E and Table S1.†

**Table 1:** Textural properties and chemical compositions of the ZBIDCs

	SA^a^ (m^2^ g^−1^)	PV_Total_^b^ (cm^3^ g^−1^)	Yield^c^ (%)	C^d^ (wt%)	H^d^ (wt%)	N^d^ (wt%)	O^d^ (wt%)	*C*_s_ e (F g^−1^)
ZBIDC-2-700	525	0.21	75	49.7	1.4	12.7	7.1	101
ZBIDC-2-800	750	0.30	62	60.0	1.8	11.2	8.3	235
ZBIDC-2-900	855	0.33	51	72.9	0.7	10.0	8.3	332
ZBIDC-2-1000	570	0.26	46	75.7	0.8	7.7	4.2	115

a Brunauer–Emmett–Teller (BET) surface area obtained from Ar isotherms at 87 K.

b Total pore volume at *P*/*P*_0_ = 0.95.

c Ratio of the vacuum dried carbon product to the benzimidazole precursor.

d Obtained by CHNO elemental analysis.

e Gravimetric specific capacitance calculated at 1 A g^−1^ and in 1 M H_2_SO_4_.

The porous carbon obtained by our synthetic route featured notably higher yields (Table 1) in comparison with other activation methods. We previously showed that the carbon obtained by KOH activation of a benzimidazole linked-polymer at a temperature of 800 °C and activator to precursor weight ratio of 2 gave only 13% yield.^48^ This is because KOH activation etches away the majority of the carbon framework to generate small pores. On the contrary, the carbon sample prepared by ZnCl_2_ activation and under similar conditions in this study (ZBIDC-2-800) offered 62% yield. The notably higher yield of carbon achieved by our present synthetic approach would be beneficial for large-scale production.

The CHNO elemental analysis (EA) and X-ray photoelectron spectroscopy (XPS) techniques were performed to evaluate the detailed elemental composition and the nature of the nitrogen species of the ZBIDCs. The results are summarized in Table 1 and S2.† The survey spectra of the ZBIDCs (Fig. S4†) displayed three pronounced signals at around 285, 399, and 532 eV, which are attributed to C 1s, N 1s, and O 1s, respectively. Due to the oxygen- and water-free nature of the chemicals and the controlled synthetic atmosphere used in this research work, the formation of oxygenated functionalities as dopants similar to what was observed in the KOH-activated carbons^49^ is highly unlikely. Consequently, the presence of oxygen peaks in the full survey spectra can be attributed to the oxidation of indium foil used as a substrate and the absorption of water from the ambient surroundings during sample preparation and handling.^19^ This claim is evidenced by the lower percentage of oxygen compared to KOH-activated carbons (up to 25 wt%) along with a small amount of indium recorded for the composition of the ZBIDCs (Table S2†). The comparable amount of nitrogen obtained by the two methods is indicative of the successful and uniform incorporation of nitrogen into the carbon matrix. Any residual mass observed when using the EA method can be justified by incomplete combustion (ash percentage in Table S2†) which is a common phenomenon for carbon materials.^40^ Furthermore, minor amounts of Zn and Cl detected by XPS are probably related to the ZnCl_2_ trapped inside closed pores.^50^ It is noteworthy that the nitrogen content obtained from the EA results was slightly higher than that from the XPS data because the latter is a surface sensitive technique while the former is a bulk analysis method. Therefore, the percentage of nitrogen obtained by EA will be used as the more reliable data for future discussion. The initial observation revealed a very high level of nitrogen doping varying in the 7.7–12.7 wt% range. The nitrogen content decreased upon increasing the activation temperature and remained unaffected when higher amounts of ZnCl_2_ were used. The ZnCl_2_-activated benzimidazole building blocks displayed noticeably higher nitrogen content when compared to their KOH-activated counterparts.^51^ For instance, under similar synthetic conditions (*T* = 700 °C and activator/precursor weight ratio = 2) ZnCl_2_-activation afforded 12.7 wt% with respect to the 5.7 wt% nitrogen doping level obtained by KOH-activation. This can be explained with the etching mechanism of the KOH activation for pore formation resulting in more carbon and heteroatom elimination during thermal treatment. To further gain insight into the evolution of the nitrogen moieties during chemical activation and carbonization, the N 1s spectra of the ZBIDCs were peak fitted and deconvoluted. In general, four main contributors (Fig. S5†) can be identified in the high-resolution N 1s of a nitrogen-doped carbon: (i) pyridinic (N-6, 398 eV), (ii) pyrrolic and/or pyridinic (N-5, 400 eV), (iii) quaternary (N-Q, 401 eV), and (iv) pyridine-*N*-oxide (N-X, 403–406 eV).^52^ The simple structure of the benzimidazole precursor consists of solely pyrrolic and pyridinic entities which are located inside the pentagonal ring.^48^ As shown in Fig. S6,† the nitrogen environments in the resulting ZBIDCs are divided into two major components: pyridinic-N (398.4 eV) and pyrrolic-N (400.4 eV). The slight impurity at higher energy (404.1 eV) is probably oxidized-N species. Interestingly, the intensity of quaternary nitrogen forms which can originate from other forms of nitrogen at elevated temperatures was low in our study, implying the higher stability of the pyrrolic and pyridinic configurations.^53^ The relative surface concentrations of the nitrogen species obtained by fitting the N 1s core level spectra are provided in Table S3.† Accordingly, the pyridinic type constituted the largest portion and the pyrrolic species comprised the second highest percentage of the total nitrogen functionalities.

### 3.3. Electrochemical performance

**Fig. 3:**
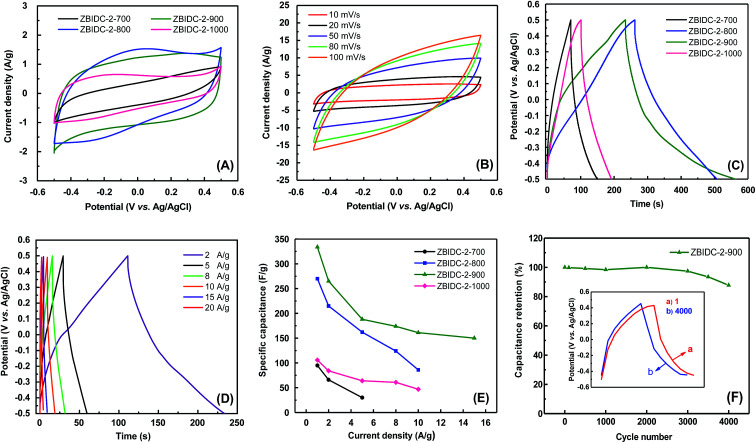
Electrochemical performance of various ZBIDC materials using a three-electrode cell in 1 M H_2_SO_4_. (A) Cyclic voltammograms at a scan rate of 5 mV s^−1^ for all ZBIDCs, (B) cyclic voltammograms of ZBIDC-2-900 at different scan rates, (C) galvanostatic charge–discharge curves at a current density of 1 A g^−1^ for all ZBIDCs, (D) galvanostatic charge–discharge curves of ZBIDC-2-900 at different current densities, (E) specific capacitance as a function of current density and (F) cyclic stability of the ZBIDC-2-900 electrode at a current density of 10 A g^−1^ over 4000 cycles (the inset shows the charge–discharge curves of the 1^st^ and 4000^th^ cycles at 10 A g^−1^).

The supercapacitive performances of the ZBIDCs samples were evaluated with a three-electrode cell configuration in 1 M H_2_SO_4_ electrolyte (Fig. 3). Due to the nearly identical porous parameters and nitrogen doping levels of the carbons formed by varying the ZnCl_2_ content, only the capacitance behavior of the carbons synthesized at different temperatures was investigated. The comparative cyclic voltammogram (CV) plots of the ZBIDCs at a scan rate of 5 mV s^−1^ are shown in Fig. 3A. The carbons prepared at temperatures of 800, 900 and 1000 °C featured nearly rectangular shapes indicating ideal electrical double-layer capacitance (EDLC) behavior. However, deviation from the ideal rectangular CV plot was observed for ZBIDC-2-700, which can be correlated to its ultrafine pores. More specifically, the narrowly distributed pores (mostly below 0.5 nm) and bottleneck morphologies obtained by activation at 700 °C are usually inaccessible to the electrolyte ions.^54,55^ In addition to the EDLC, the pseudocapacitive contribution can be recognized by the CV plots. The presence of distinct humps between −0.2 and 0.1 V is recognized as a typical signature of the pseudocapacitance contribution, which originates from redox reactions of the heteroatom functionalities on the surface of the carbon electrodes.^14^ Therefore, the total capacitive responses of the ZBIDCs can be linked to the combination of EDLC and pseudocapacitance. The CV profiles at different scan rates (Fig. 3B) retained nearly perfect symmetrical shapes, which highlights their high electrochemical stability. Increasing the scan rate to 100 mV s^−1^ led to the disappearance of the pseudocapacitance peaks and deviation from the perfect rectangular shape in the CV plots because of the limited ion transport kinetic inside the small confined pores.^17,21^

The galvanostatic charge–discharge (GCD) curves of the ZBIDCs are depicted in Fig. 3C. The GCD curves exhibited a typical isosceles triangular shape, which is an indication of electric double layer capacitive behavior. The presence of a small voltage drop (IR) is usually associated with the high equivalent series resistance (ESR) phenomenon. The slight deviation from linearity observed for all of the samples is indicative of pseudocapacitive contributions, which can be considered analogous to the humps in the CV plots. It should be noted that the GCD profiles of the ZBIDCs are not entirely symmetrical. The ZBIDC samples possess a relatively high level of heteroatom functional groups on the surface, which enhances the pseudocapacitive properties. However, some of these reactions are not reversible which causes asymmetry in the GCD graphs.^56,57^ The discharge portions of the GCD curves were used to accurately evaluate the gravimetric specific capacitance of the carbon electrodes (using eqn (1)). The gravimetric specific capacitance (*C*_s_) values for ZBIDC-2-700, ZBIDC-2-800, ZBIDC-900 and ZBIDC-2-1000 were calculated as 101, 235, 332 and 115 F g^−1^, respectively, at a current density of 1 A g^−1^ in 1 M H_2_SO_4_. To the best of our knowledge the obtained capacitance value of 332 F g^−1^ lies among the highest reported to date for different porous carbons under similar conditions.^58^ Nevertheless, the capacitance of ZBIDC-900 is lower than those of CO_2_-activated carbons using a synthetic polymer as the precursor (388 F g^−1^)^59^ and templated carbons synthesized by a two-step nanocasting process (340 F g^−1^).^54^ However, the simplicity of our synthetic protocol coupled with the availability and low cost of the precursors offers overall advantages compared to the abovementioned materials. We also investigated the performance of the optimum sample, ZBIDC-2-900, using a two-electrode configuration to ensure a fair comparison with reported porous carbons in the literature (Fig. S7†). The capacitance values (using eqn (2)) were found to be 132, 114, 79 and 62 F g^−1^ at current densities of 0.5, 1.0, 5.0 and 10.0 A g^−1^, respectively. A complete comparison between the ZBIDC-2-900 capacitive performance and recently reported porous carbons has been provided in Table S4.†

The superior electrochemical performance of the optimum sample, ZBIDC-2-900, arises from the synergistic effects of its high surface area, proper pore size distribution, sufficient degree of graphitization and high nitrogen doping level. The wider micropore size distribution of ZBIDC-2-900 compared to the other three samples is advantageous for fast ion transport through the porous network of carbon. In fact, the porous carbons composed of entirely ultrafine pores (<0.5 nm) feature higher internal resistance and poor ion diffusion whereas wide micropores facilitate ion transfer and lead to a lower internal resistance value.^60,61^ Accordingly, Yushin *et al.* showed that the optimal design of pores in a fully microporous carbon electrode benefits the rapid ion diffusion and the presence of mesopores is not required.^62^ Meanwhile, the nitrogen functional groups present on the surface of the electrode are able to modify the electron donor/acceptor nature of the carbon structure and promote an electrochemically active surface through inducing faradaic redox reactions.^63^ In particular, the basic nitrogen functionalities increase the electronic charge density of the carbon surfaces by facilitating proton adsorption when acidic solutions are used as electrolytes. The possible faradaic charge transfer reactions for the pyridinic, pyridonic and pyrrolic nitrogen configurations in acidic media are depicted in Fig. S8.†^59^ Additionally, the nitrogen sites improve the wettability of the electrodes toward aqueous electrolytes by introducing polar C–N bonds and hydrophilicity to the surface of the ZBIDCs.^64^ It has been shown that the pyrrolic and pyridinic nitrogen species located at the periphery of the carbon lattice have the most pronounced contribution to the pseudocapacitive effect,^14^ while the quaternary nitrogen entities promote the hydrophilicity and wettability of carbon.^65^ The correlations between the capacitance value and various current densities are demonstrated in Fig. 3E. Upon increasing the current density, a noticeable decrease in the capacitance of ZBIDC-2-700 and ZBIDC-2-800 was observed while ZBIDC-2-900 and ZBIDC-2-1000 featured a gradual slope change. Notably, at a high current density of 15 A g^−1^, the capacitance of ZBIDC-2-900 remained as high as 150 F g^−1^, offering an acceptable rate capability. The observed drop in capacitance upon increasing the current density is associated with the ohmic resistance caused by ion accumulation inside the narrow micropores and/or interaction between the electrolyte and surface heterogeneity or dangling bonds.^21^ Cyclic stability is regarded as a crucial aspect for evaluating the practical application of electrode materials. Accordingly, the cycling stability for the optimum carbon, ZBIDC-2-900, was evaluated by continuous GCD experiment at a relatively high current density of 10 A g^−1^ in 1 M H_2_SO_4_. As shown in Fig. 3F, ZBIDC-2-900 exhibits excellent stability with only 12.2% capacitance loss after 4000 consecutive cycles.

**Fig. 4:**
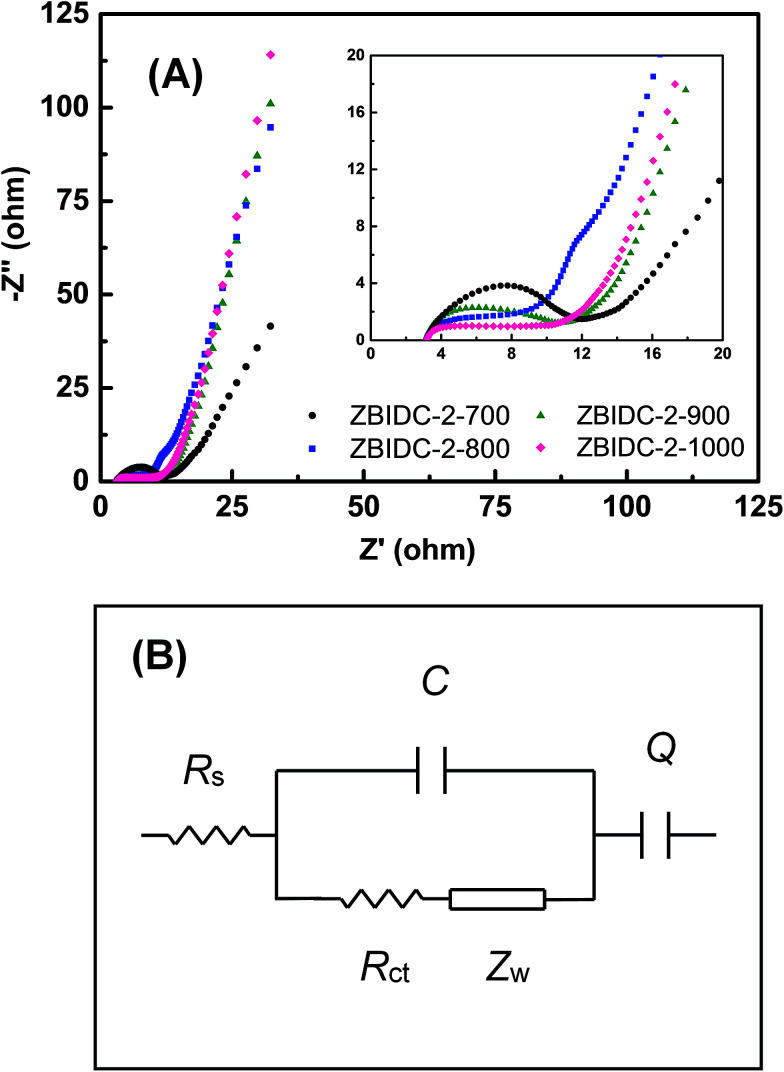
(A) Nyquist plots of the ZBIDC-based supercapacitors (the inset shows the expanded high-frequency region) and (B) the equivalent circuit model.

Electrochemical impedance spectroscopy (EIS) as a complementary method was conducted to assess the facilitated ion/electron transport way within the ZBIDC electrodes. As shown in Fig. 4A, each Nyquist impedance spectrum consists of a semicircle at the high-frequency region and a straight line in the low-frequency segment. The *Z*′ axis intercept at high-frequency represents the series resistance (*R*_s_) which includes the electrolyte resistance, intrinsic resistance of the active material, and electrical contact resistance at the interface of the active material and the current collector.^66^ The *R*_s_ values are in a range of 3 Ω for all of the samples which is probably caused by the similar ionic conductivity of the electrolyte and identical fraction of carbon black in each electrode. The relatively high *R*_s_ value is associated with limited ion accumulation inside the narrow micropores and large surface functionality (heteroatom doping). The semicircles observed at the high-frequency region of the plot (inset) represent the charge-transfer resistance (*R*_ct_) in the electrode material. A relatively small semicircle can be interpreted as a more efficient redox reaction with enhanced contact between the electrode and electrolyte.^67^ At low frequencies, the section of the tail with a slope of 45° is defined as the Warburg diffusion resistance (*Z*_w_) which features ion diffusion into the pores of the active material. The shorter length of this diffusion segment indicates lower ion diffusion resistances, which are mainly caused by the wider pore size and the enhanced surface hydrophilicity.^68^ Finally, a nearly vertical slope at the end of low-frequency region indicates a lower diffusive resistivity for the electrolyte ions within the pores of the electrode materials. The more vertical the line, the more the supercapacitor performs as an ideal capacitor.^69^ It can be understood that a short *Z*′-intercept, small radius of the semicircle and sharp slope of line featured by the Nyquist plot are characteristics of great pore accessibility for the electrolyte. The EIS spectra can be fitted by electrical equivalent circuits, which are composed of *R*_s_, *R*_ct_, *Z*_w_, *C* and *Q* (Fig. 4B). *R*_s_, *R*_ct_ and *Z*_w_ are ascribed to the series resistance, charge-transfer resistance and Warburg impedance, respectively. *C* is associated with the double layer capacitance and *Q* is correlated to the faradaic capacitance.^70^

## 4. Conclusion

Zinc chloride activated benzimidazole derived carbons (ZBIDCs) with moderate specific surface areas, optimal pore size distributions, suitable graphitization degrees and high nitrogen content were prepared by a facile, one-step, inexpensive and solvent-free synthetic procedure. The mixtures prepared by the physical mixing of benzimidazole monomers as single-source precursors (C and N) and zinc chloride as a medium-porogen were heated to high temperatures. The effective roles of ZnCl_2_ in complex formation, polymerization–carbonization and pore generation were all integrated into a single-step reaction. Adjusting the activation temperature afforded carbons with diverse textural properties and nitrogen content while varying the amount of ZnCl_2_ did not affect the physiochemical properties of the ZBIDCs. Among all of the carbons, ZBIDC-2-900 possessed the highest capacitance of 332 F g^−1^ at 1 A g^−1^ in 1 M H_2_SO_4_. This superior performance was modulated by the collaborative effects of the faradaic redox reactions, resulting from the nitrogen functional groups, and the electric double-layer capacitance (EDLC), originating from the optimal microporous structure. The excellent electrochemical results, inexpensive and commercially available precursors as well as the high yielding, solvent-free and convenient synthetic strategy highlight the bright prospects of ZBIDCs for future electrode materials in the energy storage field.

## Conflicts of interest

Authors declare no conflicts of interest.

## Supplementary Material

RA-008-C8RA00546J-s001
