# Supplementary material for: Rapid transformation of heterocyclic building blocks into nanoporous carbons for high-performance supercapacitors
Source: RSC Adv. 2018 Apr 3;8(22):12300–9. doi: 10.1039/c8ra00546j (PMC9079292; doi:10.1039/c8ra00546j)
Supplement: RA-008-C8RA00546J-s001 [file RA-008-C8RA00546J-s001.pdf]

Electronic Supplementary Material (ESI) for RSC Advances.
This journal is © The Royal Society of Chemistry 2018

Electronic Supplementary Information

Rapid transformation of heterocyclic building blocks into nanoporous carbons for high-performance supercapacitors

Babak Ashourirad,[a*] Muslum Demir,[b,c] Ryon A. Smith,[a] Ram B. Gupta[b] and Hani M. El-Kaderi [a*]

[a] Department of Chemistry [b] Department of Chemical and Life Science Engineering
Virginia Commonwealth University
Richmond, VA 23284, USA
E-mail: ashouriradb@vcu.edu, helkaderi@vcu.edu
Fax: +1 804 828 8599; Tel: +1 804 828 7505

[c]Osmaniye Korkut Ata University, Department of Chemical Engineering, 80000 Osmaniye, Turkey

Fig. S1 (A, B) Ar-87 K isotherms and corresponding PSD curves for ZBIDC-*x*-900, (C, D) N_2-77 K isotherms and corresponding PSD curves for ZBIDC-2-*y*, and (E, F) N_2-77 K isotherms and corresponding PSD curves for ZBIDC-*x*-900 (solid symbols for adsorption and empty symbols for desorption. PSD curves are offset vertically in steps of 1.0 for clarity).

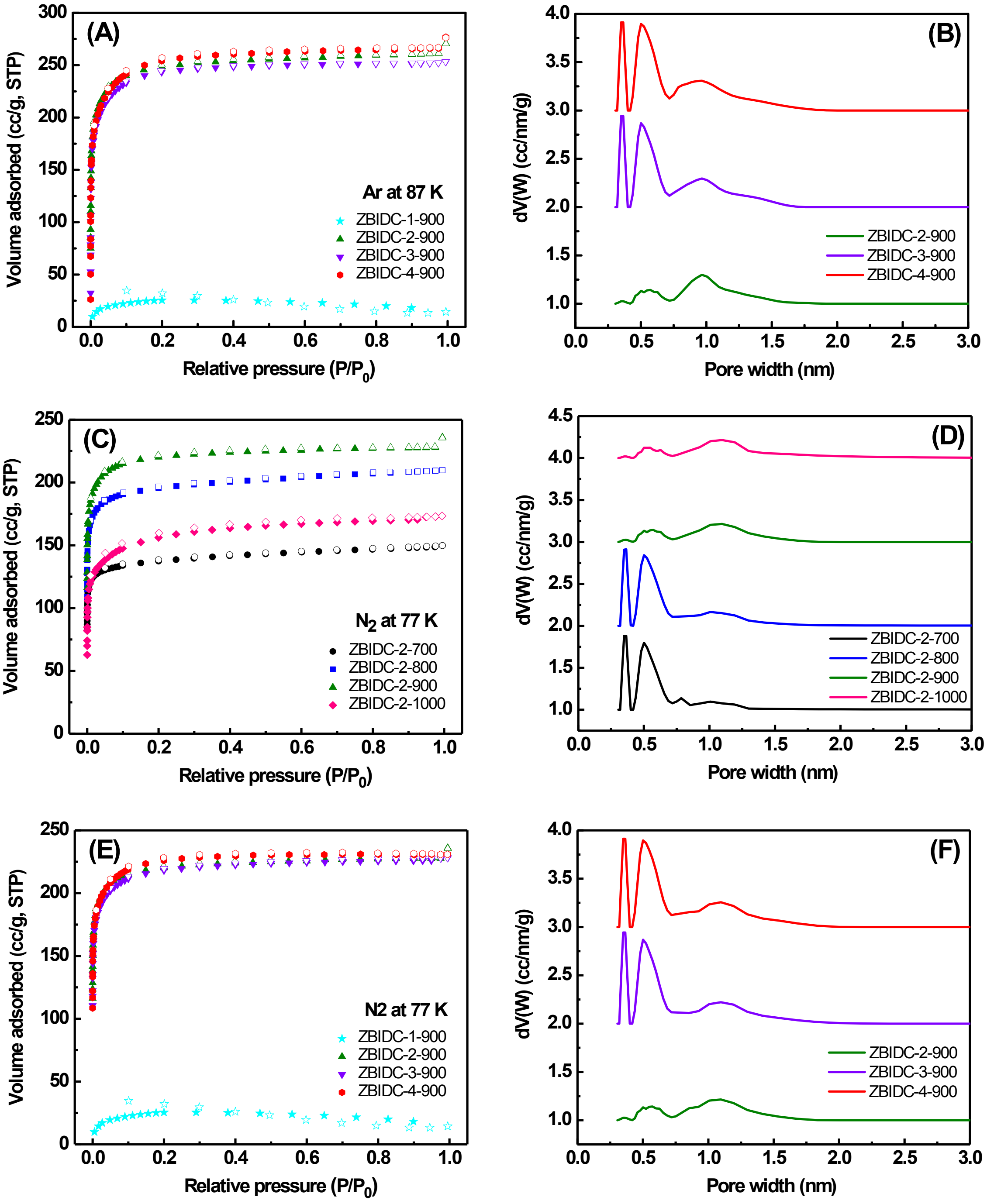

Table S1 Surface area (BET) and total pore volume (measured at P/P_0=0.95) values for ZBIDCs obtained from Ar (87 K) and N_2 (77 K) isotherms.

	Ar at 87 K		N_2 at 77 K	
	SA (m^2 g^{-1})	PV $_{Total}$ (cm^3 g^{-1})	SA (m^2 g^{-1})	PV $_{Total}$ (cm^3 g^{-1})
ZBIDC-2-700	525	0.21	545	0.23
ZBIDC-2-800	750	0.30	775	0.32
ZBIDC-2-900	855	0.33	870	0.35
ZBIDC-2-1000	570	0.26	590	0.27
ZBIDC-1-900	95	0.02	100	0.03
ZBIDC-3-900	825	0.32	855	0.35
ZBIDC-4-900	855	0.34	880	0.36

Fig. S2 BET plots for ZBIDCs from the Ar adsorption isotherms at 87 K (W = Weight of gas adsorbed at P/P_0, r = Correlation coefficient, c = C constant).

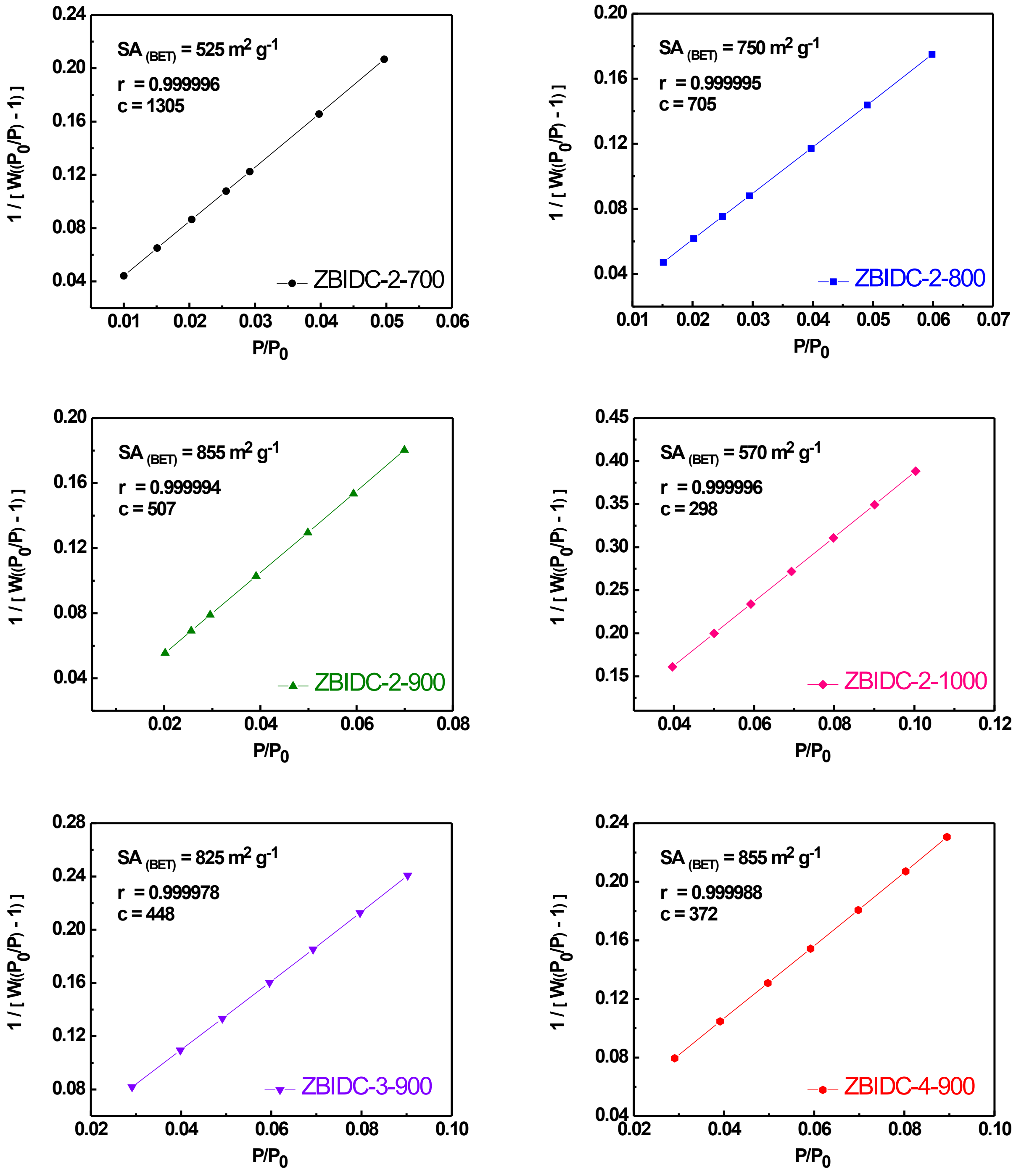

Fig. S3 BET plots for ZBIDCs from the N_2 adsorption isotherms at 77 K (W = Weight of gas adsorbed at P/P_0, r = Correlation coefficient, c = C constant).

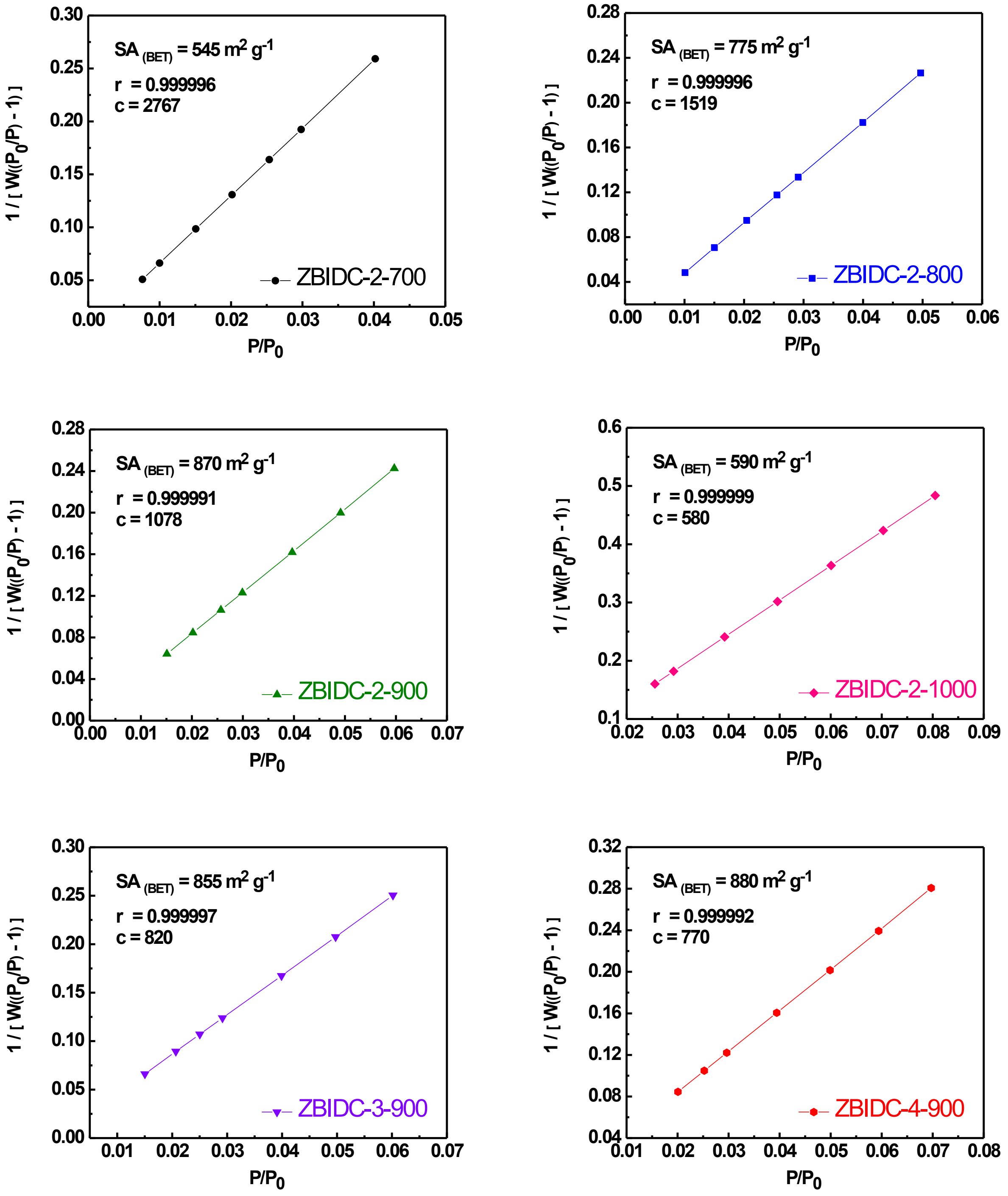

Fig. S4 X-ray photoelectron spectroscopy (XPS) survey spectra for ZBIDCs.

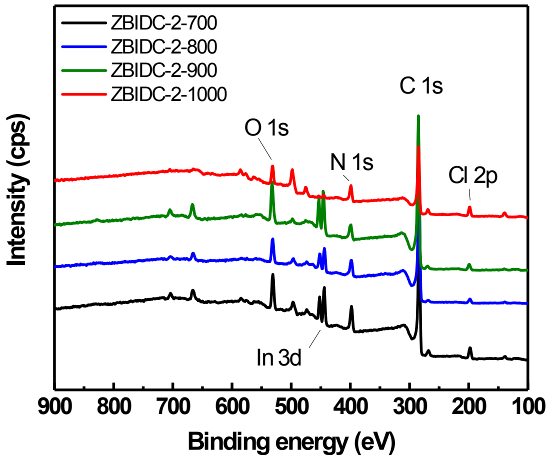

Table S2 Detailed composition of ZBIDCs by XPS and elemental analysis methods.

	XPS (At.%)						EA (Wt.%)				
	C	N	O	In	Zn	Cl	C	N	H	O	Ash
ZBIDC-2-700	75.7	10.9	8.0	0.7	1.6	3.1	49.7	12.7	1.4	7.1	29.1
ZBIDC-2-800	78.2	8.9	8.5	1.8	0.8	1.9	60.0	11.2	1.8	8.3	18.7
ZBIDC-2-900	80.3	7.3	7.8	1.0	1.3	2.3	72.9	10.0	0.7	8.3	8.1
ZBIDC-2-1000	70.7	5.5	13.3	6.1	0.7	3.8	75.7	7.7	0.8	4.2	11.6
ZBIDC-1-900	78.7	9.0	7.4	1.4	0.9	2.7	60.1	11.0	1.1	NM	NM
ZBIDC-3-900	78.8	6.7	10.5	1.9	0.6	1.6	68.3	10.4	0.9	NM	NM
ZBIDC-4-900	78.1	7.6	9.3	2.1	0.8	2.1	67.2	10.8	0.9	NM	NM

NM= Not measured

Fig. S5 The schematic representation of various nitrogen species in a typical porous carbon.

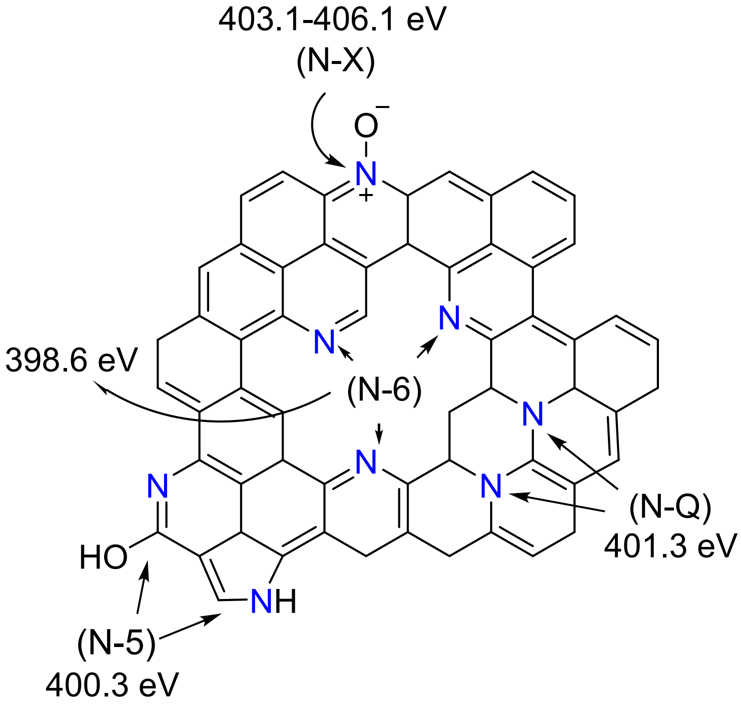

Fig. S6 High-resolution deconvoluted N 1s spectra for (A) ZBIDC-2-700, (B) ZBIDC-2-800, (C) ZBIDC-2-900, and (D) ZBIDC-2-1000.

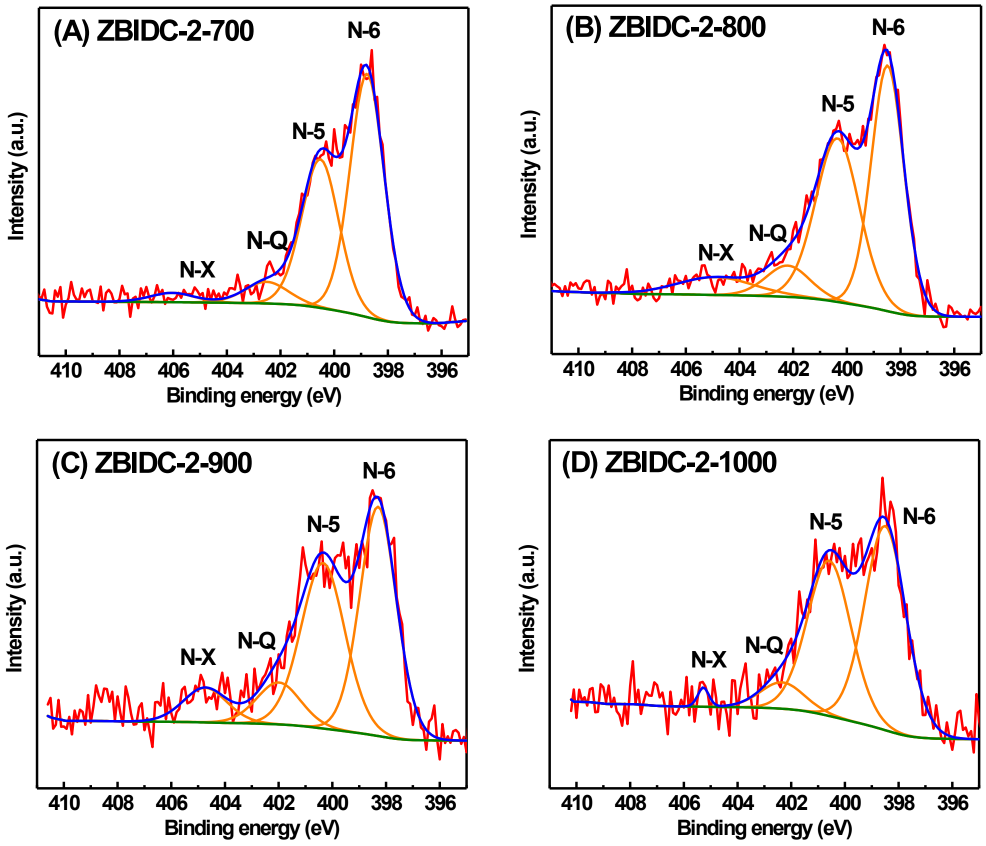

Table S3 Relative surface concentrations of nitrogen species obtained by fitting the N 1s spectra.

	N-6 (At.%)	N-5 (At.%)	N-Q (At.%)	N-X (At.%)
ZBIDC-2-700	54.6	36.5	6.2	2.7
ZBIDC-2-800	44.7	40.0	7.7	7.6
ZBIDC-2-900	44.3	38.1	9.7	7.8
ZBIDC-2-1000	50.6	41.1	7.0	1.3

Fig. S7 Electrochemical performance of ZBIDC-2-900 sample using a two-electrode cell in 1 M H_2SO_4. (A) Cyclic voltammograms of ZBIDC-2-900 at different scan rates, (B) Galvanostatic charge−discharge curves of ZBIDC-2-900 at different current densities, (C) Nyquist plot of ZBIDC-2-900 based supercapacitors

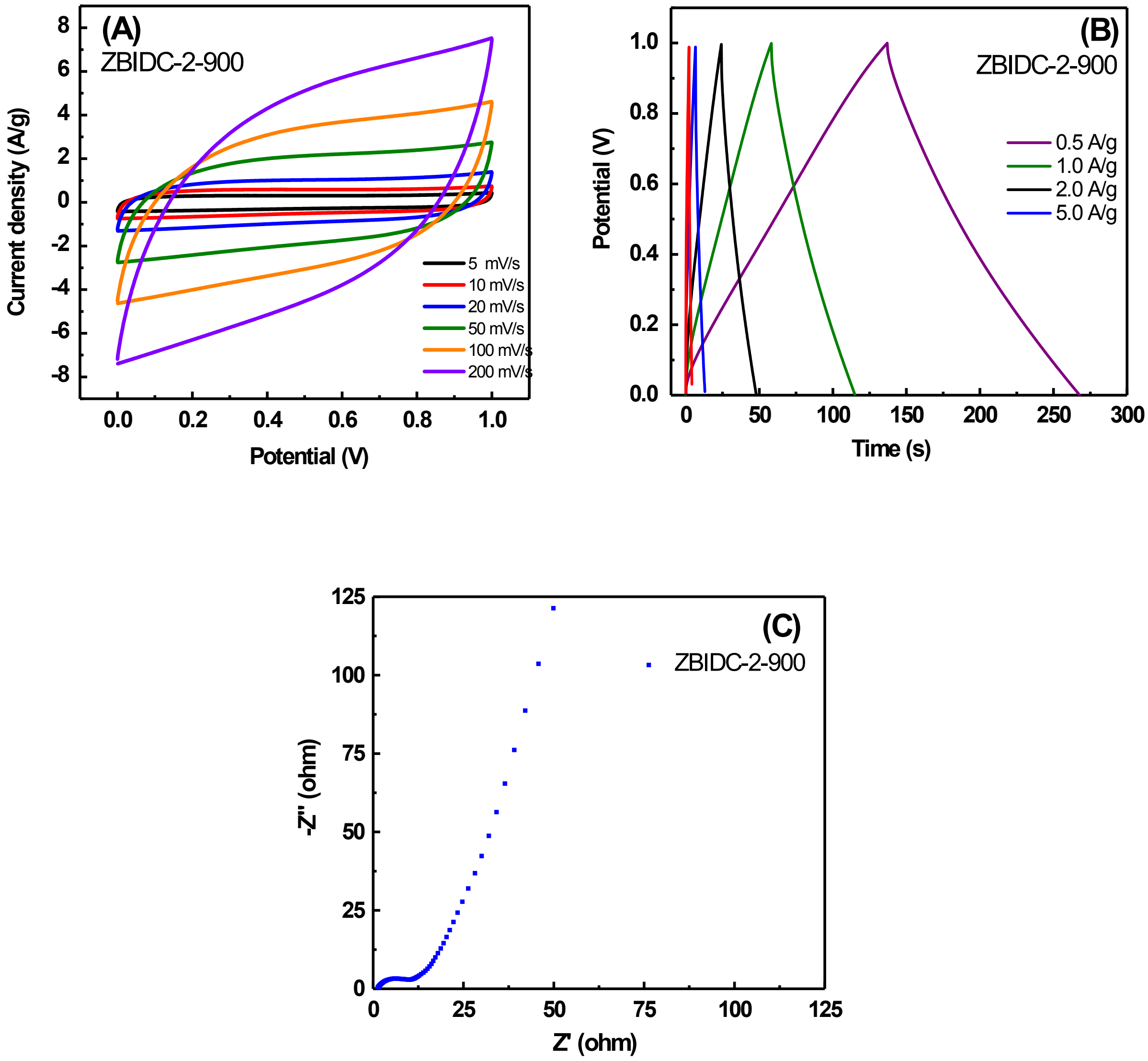

Fig. S8 Possible redox reactions related to (A) pyrrolic, (B) pyridinic, and (C) pyridonic nitrogen species in acidic media.[1]

(A)

(B)

(C)

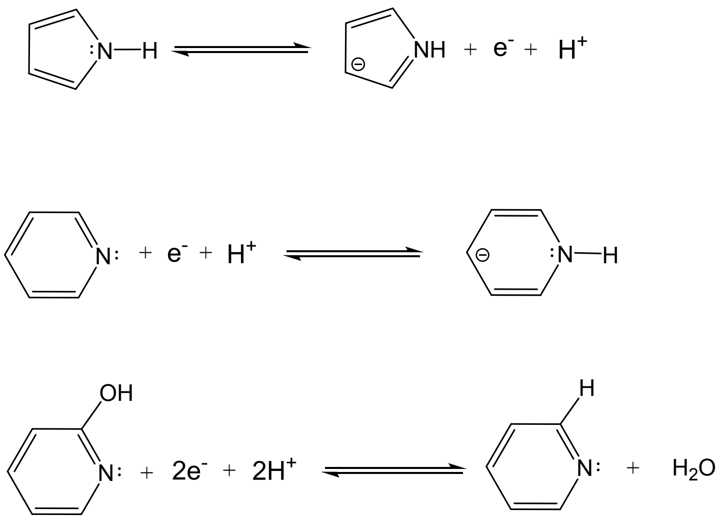

Table S4 The capacitive performance of recently reported N-doped carbons in literature (all data obtained at 1 A g^{-1} and 1 M H_2SO_4).

Materials	Capacitance (F g^{-1})		N level (Wt.%)	Reference
	three-electrode cell	two-electrode cell		
CS3-6A	388	NR	3.6	Ref [1]
N-RGO	233	NR	3.0	Ref [2]
CIRMOF-3-950	213	NR	3.3	Ref [3]
BP-800	260	NR	0.7	Ref [4]
a-NC700	296	NR	4.5	Ref [5]
NCC-1h	207	NR	9.6	Ref [6]
BAX-M	236	NR	5.9	Ref [7]
Y-AN	340	NR	6.0	Ref [8]
CA-GA-2	250	NR	4.4	Ref [9]
HPC3-600	300	NR	2.7	Ref [10]
1g:3g 600	NR	240	0	Ref [11]
NMC50	NR	290	8.4	Ref [12]
PNCMs	NR	200	4.2	Ref [13]
C3	NR	220	0	Ref [14]
ZBIDC-2-900	332	115	10.0	This work

NR = Not reported

References

1. N. P. Wickramaratne, J. Xu, M. Wang, L. Zhu, L. Dai and M. Jaroniec, *Chem. Mater.*, 2014, **26**, 2820-2828.
2. Y.-H. Lee, K.-H. Chang and C.-C. Hu, *J. Power Sources*, 2013, **227**, 300-308.
3. J.-W. Jeon, R. Sharma, P. Meduri, B. W. Arey, H. T. Schaef, J. L. Lutkenhaus, J. P. Lemmon, P. K. Thallapally, M. I. Nandasiri, B. P. McGrail and S. K. Nune, *ACS Appl. Mater. Interfaces*, 2014, **6**, 7214-7222.
4. H. Zhu, J. Yin, X. Wang, H. Wang and X. Yang, *Adv. Funct. Mater.*, 2013, **23**, 1305-1312.
5. L. Wang, Z. Gao, J. Chang, X. Liu, D. Wu, F. Xu, Y. Guo and K. Jiang, *ACS Appl. Mater. Interfaces*, 2015, **7**, 20234-20244.
6. L. Li, Q. Zhong, N. D. Kim, G. Ruan, Y. Yang, C. Gao, H. Fei, Y. Li, Y. Ji and J. M. Tour, *Carbon*, 2016, **105**, 260-267.
7. M. Seredych, D. Hulicova-Jurcakova, G. Q. Lu and T. J. Bandosz, *Carbon*, 2008, **46**, 1475-1488.
8. C. O. Ania, V. Khomenko, E. Raymundo-Piñero, J. B. Parra and F. Béguin, *Adv. Funct. Mater.*, 2007, **17**, 1828-1836.
9. L. Zhao, L.-Z. Fan, M.-Q. Zhou, H. Guan, S. Qiao, M. Antonietti and M.-M. Titirici, *Adv. Mater.*, 2010, **22**, 5202-5206.
10. J.-S. Wei, H. Ding, Y.-G. Wang and H.-M. Xiong, *ACS Appl. Mater. Interfaces*, 2015, **7**, 5811-5819.
11. C. Wang, M. J. O'Connell and C. K. Chan, *ACS Appl. Mater. Interfaces*, 2015, **7**, 8952-8960.
12. X. Yang, C. Li and R. Fu, *J. Power Sources*, 2016, **319**, 66-72.
13. Y.-Y. Wang, B.-H. Hou, H.-Y. Lu, F. Wan, J. Wang and X.-L. Wu, *RSC Adv.*, 2015, **5**, 97427-97434.
14. A. Jain, C. Xu, S. Jayaraman, R. Balasubramanian, J. Y. Lee and M. P. Srinivasan, *Microporous Mesoporous Mater.*, 2015, **218**, 55-61.
